# A heating method for producing frozen pizza ingredients with increased total polyphenol content and 2,2‐diphenyl‐1‐picrylhydrazyl radical scavenging activity

**DOI:** 10.1002/fsn3.598

**Published:** 2018-02-20

**Authors:** Lennie K. Y. Cheung, Haruo Tomita, Toshikazu Takemori

**Affiliations:** ^1^ Energy Technology Laboratories Osaka Gas Co., Ltd. Osaka Japan

**Keywords:** antioxidant capacity, heating, Maillard reaction, pizza, processed vegetables, total polyphenol content

## Abstract

Despite growing demand for more healthful frozen pizza, current technologies for increasing potential healthfulness such as reformulation or enrichment of raw ingredients may lead to undesirable changes in the final product. This study evaluated alternative heat treatments of selected frozen pizza ingredients as methods for increasing the healthfulness of frozen pizza. Four common vegetable toppings (i.e., onion, corn, Japanese green pepper, and red pepper) were heated on a 250°C hot plate, and commercially available par‐baked pizza base was reheated at 500°C for 50 s to induce browning. These alternatively heat‐treated (AHT) ingredients were compared to their conventional counterparts (e.g., steam‐blanched vegetable toppings and commercially available par‐baked pizza base, respectively) in terms of total polyphenol content (TPC) and 2,2‐diphenyl‐1‐picrylhydrazyl radical scavenging activity (DPPH RSA). TPC increased and was correlated with internal temperature for onion and peppers during alternative heat treatment, while increases in DPPH RSA of AHT onion and pizza base may be due to the formation of Maillard reaction products. Replacing conventional samples with AHT counterparts increased TPC and DPPH RSA by 1.2‐fold to 1.6‐fold and 1.3‐fold to 2.1‐fold, respectively, for vegetable toppings after reheating at 230°C for 12 min. Significant differences in acceptability of sensory attributes (i.e., appearance, taste, aroma, texture, and overall preference) were not observed between AHT and conventional vegetable topping when incorporated into pizza. These results suggest that alternative heat treatment of raw ingredients, particularly vegetable toppings, for the purpose of increasing TPC and DPPH RSA may be a viable method for increasing the potential healthfulness of frozen pizza.

## 1 INTRODUCTION

Increasing consumer demand for convenient foods continues to drive growth in the global frozen food market. Frozen ready meals account for over 30% of this market, which had a total value of USD 250 billion in 2015 and was forecasted to undergo significant expansion in the future (Grand View Research, 2016). Although consumption of frozen pizza remains high, increasing consumer demand for more healthful options coupled with increasing competition from the food service sector has prompted manufacturers to develop more healthful pizzas (Bartelme, 2016). One method of boosting pizza healthfulness is increased use of vegetables, a tactic commonly employed in the food service sector (Bartelme, 2016). A diet high in vegetables (i.e., five or more servings per day) may reduce the risk of all‐cause mortality (Wang et al., 2014), and plant‐based polyphenols in particular have been associated with health‐enhancing effects due in part to their antioxidant activity (Shahidi & Ambigaipalan, 2015).

Raw vegetables typically undergo thermal blanching prior to their incorporation into processed foods. Although conventional thermal blanching processes such as steaming and boiling are effective at preserving fresh attributes (e.g., bright colors and structural integrity) and decreasing microbial load, blanching‐induced tissue softening has been reported to result in losses of both polyphenols and antioxidant capacity (Castro et al., 2008; Chuah et al., 2008; Myojin et al., 2008; Puupponen‐Pimiä et al., 2003). In contrast, heating various vegetables by methods not typically used for blanching such as oven‐baking, stir‐frying, or microwaving may yield products with polyphenol contents or antioxidant capacity that remain comparable or even exceed their raw counterparts (Chuah et al., 2008; Uchida, Tomita, Takemori, & Takamura, 2017; Yamaguchi et al., 2001). It is plausible, then, that the aforementioned cooking processes could be used as methods for increasing bioavailable polyphenol content and potential antioxidant capacity in processed vegetables. To our knowledge, this relationship has yet to be exploited in the processed food industry.

Pizza base is the largest component of pizza by weight and may also be a viable medium of increasing the healthfulness of frozen pizza. Reformulation of dough to include healthful ingredients such as plant polyphenols has been shown to result in breads with higher antioxidant capacity (Altunkaya, Hedegaard, Brimer, Gökmen, & Skibsted, 2013; Sivam, Sun‐Waterhouse, Waterhouse, Quek, & Perera, 2011). However, such methods can alter the sensory properties of the final product, require testing for appropriate dosages to minimize the risk of toxicity, and may result in the formation of indigestible complexes and reduction of free amino acid contents (Altunkaya et al., 2013; Sivam et al., 2011; Świeca, Gawlik‐Dziki, Dziki, Baraniak, & Czyż, 2013). A simpler method for increasing the healthfulness of pizza bases may be to adjust the par‐baking conditions. Specifically, higher temperatures and longer baking times have been shown to yield pizza bases with higher antioxidant capacity (Moore, Luther, Cheng, & Yu, 2009), likely resulting from the formation of Maillard reaction products (Michalska, Amigo‐Benavent, Zielinski, & del Castillo, 2008; Morales, Martin, Açar, Arribas‐Lorenzo, & Gökmen, 2009). Maillard reaction products formed during high temperature heating of bakery products have been shown to have antioxidant capacity *in vitro*, and exert health‐enhancing effects such as reducing lipid oxidation and antihypertensive activity in rats (Patrignani, Rinaldi, & Lupano, 2016). A method of increasing potential healthfulness without reformulation has great industrial value as it would allow pizza base manufacturers to improve their established products without changing their current dough recipes.

The objective of this study was to determine whether current conventional heat processing methods could be altered to yield foodstuffs with higher potential healthfulness, and whether these products could increase the potential healthfulness of frozen pizzas if used in lieu of conventional ingredients. Specifically, four types of common vegetable pizza topping materials—namely onion (*Allium cepa*), Japanese green pepper, and red pepper (*Capsicum annuum* L. var. *grossum)* and corn—were heated by hot plate as an alternative to steam blanching, while par‐baked pizza bases were heated again to induce the formation of Maillard reaction products. These products, as well as assembled pizza consisting of such ingredients, were assessed for total polyphenol content, DPPH radical scavenging activity, and acceptability of sensory attributes.

## 2 METHODS AND MATERIALS

Methanol was obtained from Wako Pure Chemicals Industries, Ltd. (Osaka, Japan); acetone and sodium carbonate from Kanto Chemical Co., Inc. (Tokyo, Japan); and Folin & Ciocalteu's phenol reagent, gallic acid, (±)‐6‐hydroxy‐2,5,7,8‐tetramethyl‐chromane‐2‐carboxylic acid (Trolox), and 2,2‐Diphenyl‐1‐picrylhydrazyl (DPPH) reagent from Sigma‐Aldrich (St. Louis, MO, USA). Food materials were purchased locally.

### 2.1 Sample preparation

#### 2.1.1 Vegetable pizza toppings

Onions, Japanese green pepper, and red pepper were washed, diced by hand into pieces of ca. 7 × 7 mm, patted dry with a paper towel and used for heat treatments on the same day. Frozen corn‐off‐the‐cob (Top Valu, Chiba, Chiba, Japan) was thawed and brought to room temperature, patted dry, and used as is. A single layer of cooking sheet (12 μm aluminum foil with silicon coating on the food contact surface) was preheated on top of a hot plate with a surface area of 25 × 25 cm (Ninos ND‐2, As One Corporation, Osaka, Osaka, Japan) and set to 250°C, upon which 15 g of vegetable was spread in a single layer (i.e., with each food piece being in direct contact with the cooking sheet) to commence the alternative heating process. Vegetables were heated without agitation under a lid for increasing intervals of 30 s until burnt aromas could be detected, at which point the samples were considered to be unfit for use as frozen ingredients. Three replicates were prepared for each time interval. The maximum heating times for onion, corn, Japanese green pepper, and red pepper were 2, 2.5, 2, and 2.5 min, respectively. Vegetable toppings heated on the hot plate to maximum heating times are hereinafter referred to as the alternatively heat‐treated (AHT) vegetable topping materials. Conventional (i.e., steam‐blanched) vegetable toppings were prepared by steaming 15 g of diced onion, Japanese green pepper, or red pepper for 3 min (Healsio Oven AX‐SP1, Sharp Corporation, Sakai, Osaka, Japan). Upon removal from the heating environment, samples were weighed, frozen at −18°C, lyophilized and ground. Commercial frozen corn was used as is to represent conventional frozen corn. The moisture content of raw and heated vegetable samples was determined gravimetrically.

#### 2.1.2 Pizza base

AHT pizza bases were prepared by heating a commercial, par‐baked pizza base (Top Valu, Chiba, Chiba, Japan) in a 500°C electric oven (F0810 Electric Furnace, Yamato Scientific Co., Ltd., Tokyo, Japan) for 50 s. The crust and crumb of the commercial pizza base (i.e., the conventional product) before and after alternative heat treatment were separated manually, lyophilized, and ground prior to chemical analyses.

#### 2.1.3 Assembled pizza

Two types of assembled (i.e., multi‐ingredient) frozen pizzas were prepared for reheating. Conventional frozen pizza was prepared by placing ca. 50 g of each frozen conventional vegetable topping on commercially available par‐baked pizza base. AHT pizza was prepared by placing ca. 50 g of each frozen AHT vegetable topping on top of a frozen AHT pizza base. Both types of pizzas were subjected to reheating in a gas oven (230°C) for 12 min. These conditions were selected based on those recommended for commercially available frozen pizzas and were applied to represent consumer processing prior to consumption. Triplicate samples were prepared and reheated per pizza type. Reheated pizza samples were promptly frozen, and each ingredient manually separated before lyophilizing and grinding. Moisture content of reheated samples was determined gravimetrically.

### 2.2 Temperature measurement of vegetable toppings

The change in internal temperature of one piece of vegetable per 15 g sample during steaming or hot plate heating was measured by manual insertion of a 0.5 mm diameter K‐type thermocouple (Hayashi Denko Co., Ltd., Tokyo, Japan) into a randomly selected piece of vegetable. Temperature data were recovered from a desktop computer connected to the thermocouples via a signal conditioner (Model M6DBS‐08P, M‐System Co., Ltd., Osaka, Osaka, Japan). Temperature measurement was repeated for each type of vegetable topping until consistent readings over at least three replicates were obtained to ensure that the temperature readings were accurate and reproducible.

### 2.3 Visual comparison of pizza bases

Visual comparison of the crumb and crust of AHT and commercial pizza bases were conducted at ambient temperature after cutting test samples into slices of ca. 3 mm thickness, and observing under a digital microscope (VHX‐2000, Keyence Corporation, Tokyo, Japan).

### 2.4 Sample extraction

Extraction of vegetable topping materials for chemical analyses was conducted based on the methods described by Kähkönen et al. (1999). Lyophilized samples (250 mg for Japanese green pepper, and 500 mg for onion, corn, and red pepper) were weighed into a centrifuge tube, into which 10 ml of 80% aqueous methanol was added. Samples were then sonicated at ambient temperature for 10 min, followed by centrifugation at 15,000 *g* for 15 min at 6°C. The supernatant was collected and, with the exception of corn, the extraction process repeated two more times. Corn samples were extracted for a total of two times. The pooled supernatant was used as is for chemical analysis. Pizza bases were extracted using an adaptation of the method described by Moore et al. (2009). Ground samples (1.25 g) were mixed with 10 ml of 50% acetone, and the suspension lightly stirred before sonication (15 min) and centrifugation at 15,000 *g* for 15 min at 6°C. The supernatant was collected, and the extraction repeated with another 10 ml of extraction solvent. Pooled supernatant was used in subsequent chemical analyses. All samples were extracted in triplicate and on the same day as chemical analyses.

### 2.5 Total polyphenol content

Total polyphenol content (TPC) of vegetable topping materials was determined as described by Kähkönen et al. (1999), with slight modifications. Standard or diluted sample extract (160 μl) was mixed with 800 μl of Folin‐Ciocalteu reagent (diluted by a factor of 10 using deionized water). This mixture was stirred, and 640 μl of sodium carbonate (7.5%) was added within 8 min. The absorbance of this sample at 765 nm was measured after 30 min of incubation at ambient temperature. The TPC of pizza base samples was determined based on the method described in Moore et al. (2009). Briefly, 100 μl of Folin‐Ciocalteu reagent, 300 μl of 20% sodium carbonate, and 1.2 ml of deionized water were sequentially added to 20 μl of standard or sample extract. After incubation (2 h at ambient temperature), the absorbance of this mixture was measured at 765 nm using a spectrophotometer (UV 1800 UV spectrophotometer, Shimadzu Corporation, Kyoto, Kyoto, Japan). Triplicate assay samples were prepared for each sample. A gallic acid standard curve was prepared each day of analysis, and TPC was expressed as milligram of gallic acid equivalents per gram of dried sample (mg GAE/g dw).

### 2.6 DPPH radical scavenging activity

Antioxidant capacity of vegetable topping materials was determined as 2,2‐Diphenyl‐1‐picrylhydrazyl radical scavenging activity (DPPH RSA) using a method adapted from that described by Turkmen, Sari, and Velioglu (2005). Working solutions of 0.1 mM DPPH solution were prepared on the day of analysis from a 0.5 mM DPPH stock solution prepared in methanol. Standards or diluted sample extracts (400 μl) were mixed with 1.2 ml of 0.1 mM DPPH working solution. These samples were left to incubate in the dark for 60 min at ambient temperature, after which their absorbance at 517 nm was measured. The DPPH RSA of pizza base samples was evaluated based on the method described in Cheng, Moore, and Yu (2006) with slight modification. Briefly, 800 μl of DPPH working solution (0.208 mM prepared in 50% acetone) was mixed with 800 μl of sample, and incubated in the dark for 40 min at ambient temperature before having its absorbance read at 515 nm. Triplicate assay samples were prepared for all samples. A Trolox standard curve was prepared on each day of analysis, and DPPH RSA was expressed as micromole of Trolox equivalents per gram dried sample (μmol Teq/g dw).

### 2.7 Color measurement

Ground samples (ca. 350 mg) were weighed into a clean Petri dish (25 mm diameter) prior to color measurement using a spectrophotometer (CM‐5 Spectrophotometer, Konica Minolta, Inc., Tokyo, Japan). Triplicate readings were obtained for each sample.

### 2.8 Sensory evaluation

The acceptability of AHT vegetable toppings relative to their conventional counterparts was evaluated using the 9 point hedonic scale described by Peryam and Pilgrim (1957). Eleven panelists were recruited from the Energy Technology Laboratories of Osaka Gas Co., Ltd. in Osaka, Japan. The number of panelists met the required number for subsequent data analysis using Type I and II errors of 5% and 10%, respectively, and assuming a RMSL (quotient of root mean square error and scale length) of 0.14 and a difference of 1.6 between means to indicate significant difference (Hough et al., 2006). The untrained panel was composed of six females and five males between the ages of 22 and 72 years. One panelist was Caucasian, and the rest were of Asian descent. Conventional or AHT frozen vegetable topping (12 g) was individually reheated on commercially available par‐baked pizza base with 20 g of pizza sauce (Megmilk Snow Brand Co., Ltd., Tokyo, Japan), and 50 g of shredded gouda cheese (Top Valu, Chiba, Chiba, Japan). These pizzas were frozen overnight, reheated in a gas oven at 230°C for 10 min, and kept at 65°C until serving. Panelists were instructed to evaluate food samples one at a time and indicate their preference for appearance, taste, aroma, texture, and overall preference.

### 2.9 Data analysis

One‐way analysis of variance and Tukey's differentiation (*p *≤* *.05) were performed using SPSS 15 on TPC, DPPH RSA, and color parameters with heating time as the factor. Pearson correlation coefficient and its associated significance (*p *≤* *.05) were calculated in Microsoft Excel using the *tdist* function to evaluate relationships between internal temperature (at every 30 s interval of heating time) and TPC, DPPH RSA, or color parameters; color parameters and DPPH RSA; and TPC and DPPH RSA. The TPC and DPPH RSA of conventional or AHT pizza samples after reheating were estimated per 12 g (wet weight) of each vegetable topping and 140 g of pizza crust. Comparison of sensory attributes between conventional and AHT vegetable toppings and comparison of TPC and DPPH RSA between conventional or AHT pizza bases were performed using paired and equal variance *t*‐tests on Microsoft Excel, respectively.

## 3 RESULTS AND DISCUSSION

### 3.1 TPC and DPPH RSA of AHT and conventional pizza ingredients

**Figure 1:**
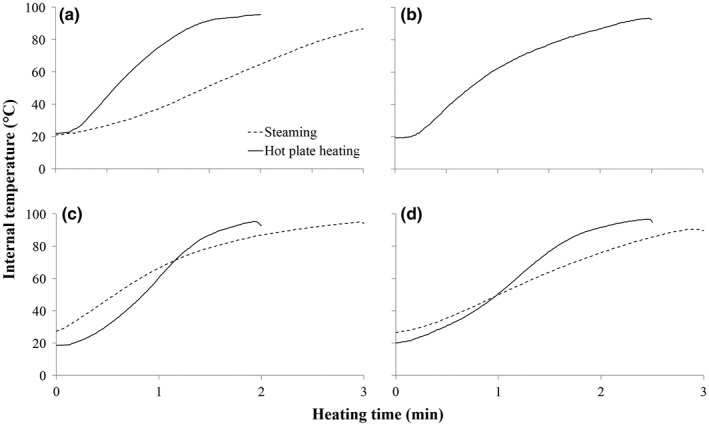
Change in mean internal temperature (*n *=* *3) of diced onion (a), thawed corn‐off‐the‐cob (b), diced Japanese green pepper (c), and diced red pepper (d) during steaming (dashed line) or hot plate heating (unbroken line)

**Figure 2:**
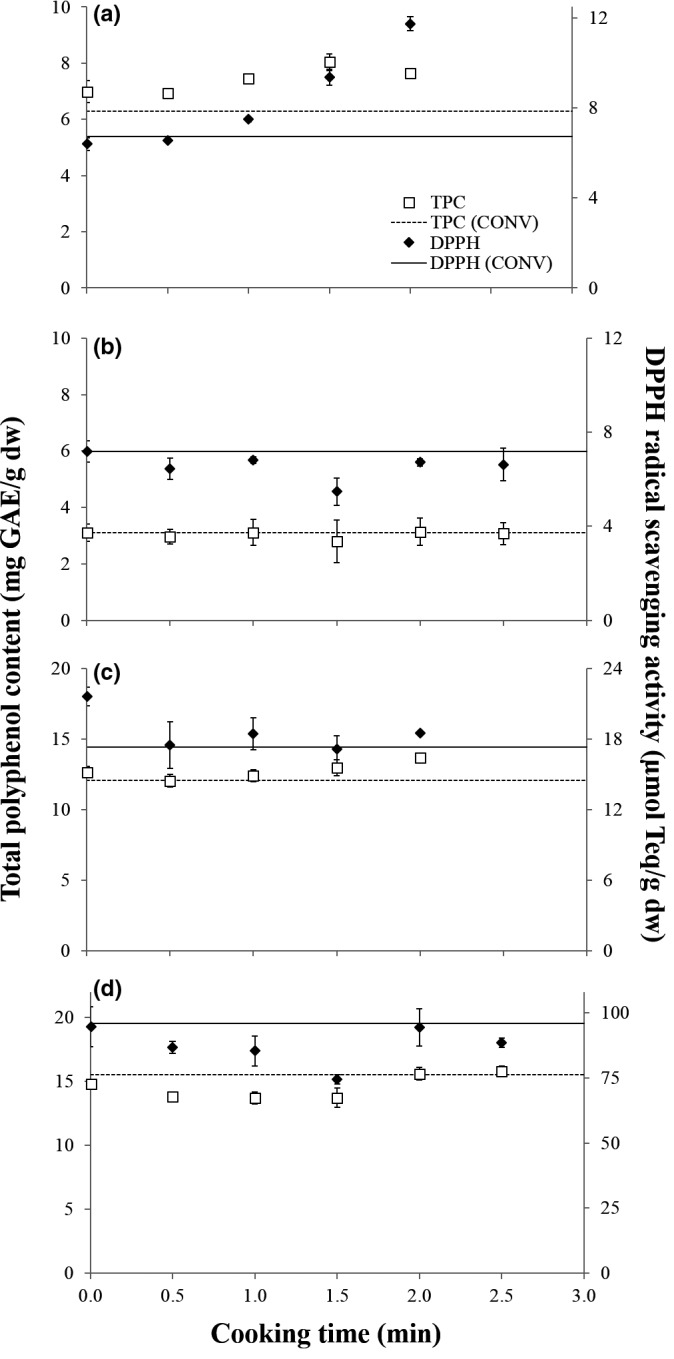
Total polyphenol content (mg GAE/g dw) and DPPH radical scavenging activity (μmol Teq/g dw) of diced onion (a), thawed corn‐off‐the‐cob (b), diced Japanese green pepper (c), and diced red pepper (d) heated on a 250°C hot plate for up to 2.5 min. The total polyphenol content and DPPH radical scavenging activity of conventional (CONV) samples are shown as a dashed or unbroken line, respectively, in each graph. Conventional samples were prepared by steam blanching for 3 min (onion and peppers) or used as is after thawing (frozen corn‐off‐the‐cob)

The change in internal temperature of vegetable toppings during steam blanching or hot plate heating is shown in Figure 1. Internal temperatures of vegetable pieces increased more rapidly when heated by hot plate and reached similar, if not higher, final temperatures than vegetable pieces steamed for 3 min (i.e., typical blanching conditions). Changes in TPC and DPPH RSA throughout hot plate heating depended on the type of vegetable topping (Figure 2) and are discussed separately below.

As shown in Figure 2a, onion TPC increased during the first 1.5 min of hot plate heating before decreasing slightly, with AHT onion having significantly higher TPC than raw onion and both samples having significantly higher TPC than conventional onion (7.6 ± 0.1, 7.0 ± 0.4, and 6.3 ± 0.3 μmol Teq/g dw, respectively). The decrease in TPC after steaming may be in part due to oxidation by polyphenol oxidase, the activity of which may persist during the initial heating of vegetables (Uchida et al., 2017). The TPC of raw onion measured falls within the range (2.5–24.49 mg GAE/g dw) of those reported in other studies (Gorinstein et al., 2008; Kähkönen et al., 1999).

TPC of corn samples remained relatively stable throughout hot plate heating and was the lowest of the vegetable toppings evaluated in this study (Figure 2b). Although thermal processing has been reported to increase free phenolic and free ferulic acid content in sweet corn (Dewanto, Wu, & Liu, 2002), the lack of increase observed in this study may be explained by the use of a commercial frozen product for experimentation. Should the processing of this product include heat treatments such as blanching, the subsequent heat treatment employed in this study would be less likely to induce observable changes in TPC.

Figures 2c and 2d show the TPC of heated Japanese green pepper and red pepper, respectively. AHT Japanese green pepper had significantly higher TPC than its conventional counterpart (13.7 ± 0.2 and 12.1 ± 0.7 mg GAE/g dw, respectively), but both contents were statistically similar to raw samples (12.7 ± 0.4 mg GAE/g dw). In contrast, raw, AHT, and conventional red pepper samples all had statistically similar TPC of 14.8 ± 0.3, 15.8 ± 0.4, and 15.5 ± 0.4 mg GAE/g dw, respectively. TPC of both pepper types was found to first decrease before increasing as heating progressed (i.e., after hot plate heating of 0.5 and 1.5 min for Japanese green and red peppers, respectively). Decrease in TPC at the start of heating may be attributed to the enzymatic degradation of polyphenols by polyphenol oxidase, and the delayed increase of red pepper TPC relative to Japanese green pepper TPC could be explained by the higher thermal stability of red pepper polyphenol oxidase (Castro et al., 2008; Uchida et al., 2017). The TPC of peppers in this study was lower than that of red pepper (52.27 ± 2.03 mg GAE/g dw), as reported by Chen and Kang (2013). This discrepancy may be explained by the inclusion of bound and esterified polyphenols in their quantification, and differences in sample preparation (e.g., red peppers were dried at 40°C in the aforementioned study but lyophilized in the current study) (Chen & Kang, 2013). Of the various polyphenols found in bell peppers including cinnamic acids and flavonoids such as luteolin, high contents of quercetin derivatives in particular were associated with the *in vitro* antioxidant activity and cellular anticancer activity exhibited by pepper extracts (Jeong et al., 2011).

In this study, TPC was measured using an adaptation of the Folin–Ciocalteu assay described by Singleton and Rossi (1965), a method commonly used for quantifying phenolics in plant foods due to its simplicity and economic feasibility (Bastola, Guragain, Bhadriraju, & Vadlani, 2017; Kähkönen et al., 1999; Sánchez‐Rangel, Benavides, Basilio Heredia, Cisneros‐Zevallos, & Jacobo‐Velázquez, 2013). While the widespread use of this method for quantifying TPC allows for comparison of values across studies, there has been growing concern regarding the accuracy of the data obtained. For example, the presence of compounds such as ascorbic acid has been shown to overestimate polyphenolic content (Bastola et al., 2017; Sánchez‐Rangel et al., 2013). Therefore, it is possible that the TPC reported above for raw onion and pepper samples may be an overestimation of true contents. Conventional corn samples may be less prone to overestimation, as ascorbic acid contents were found to decrease upon blanching and frozen storage (Myojin et al., 2008; Puupponen‐Pimiä et al., 2003). Heated samples may also be less prone to interference, as ascorbic acid contents in vegetables have been shown to decrease upon heating (Castro et al., 2008; Dewanto et al., 2002; Vega‐Gálvez et al., 2009).

AHT onion exhibited almost twofold the DPPH RSA of raw onions (11.8 ± 0.3 and 6.4 ± 0.3 μmol Teq/g dw, respectively) (Figure 2). Antioxidant activity of raw onions has been attributed to polyphenols such as quercetin glycosides (Gorinstein et al., 2008; Yamaguchi et al., 2001), while ascorbic acid has been reported to account for less than 5% of overall antioxidant activity (Chu, Sun, Wu, & Liu, 2002). Indeed, a significant correlation between TPC and DPPH RSA during the hot plate heating of onion was found (*r *=* *0.7372, *p *=* *.0112).

Similar to TPC, DPPH RSA remained relatively stable in corn throughout hot plate heating, the absolute values of which were lowest of the vegetables evaluated in this study (Figure 2). An increase in antioxidant activity of sweet corn after heat processing was reported by Dewanto et al. (2002) and thought to be attributed to dissolution of bound ferulic acid, while ascorbic acid accounted for only 1.5% of total antioxidant activity.

Pepper samples generally had lower DPPH RSA after heating (Figure 2). The largest decrease in activity was observed in red pepper samples heated on the hot plate for 1.5 min, which only exhibited 79% of the activity of raw red peppers. These findings suggest that at least some of the compounds contributing to antioxidant capacity in raw peppers were heated labile. One such antioxidant may be ascorbic acid, which has been reported to account for 23–91.2% of RSA in selected raw pepper varieties (Chu et al., 2002; Chuah et al., 2008; Yamaguchi et al., 2001), but decrease in contents by 55–70% in whole peppers after thermal blanching and up to 98.2% after drying at 90°C in cut red peppers (Castro et al., 2008; Vega‐Gálvez et al., 2009).

Pearson correlation coefficients between internal temperature and TPC or DPPH RSA of vegetable toppings during heating are shown in Table 1. With the exception of corn, the TPC of all vegetable toppings had significant (onion and Japanese green pepper) or near significant (red pepper) positive correlation with internal temperature. As discussed above, this phenomenon may be attributed to the heat‐induced dissociation of previously bound polyphenols, which may lead to a change in the quantity and quality of antioxidative compounds, coupled with heat inactivation of polyphenol‐degrading enzymes (Dewanto et al., 2002; Uchida et al., 2017).

**Table 1:** Pearson correlation coefficients and their significance between total polyphenol content or DPPH radical scavenging activity and internal temperature^a^ during alternative heat treatment (i.e., hot plate heating) of onion, corn, Japanese green pepper, and red pepper

Vegetable toppings	Pearson correlation coefficients^b^ with internal temperature (*p*‐value)	
	Total polyphenol content (mg GAE/g dw)	DPPH radical scavenging capacity (μmol Teq/g dw)
Onion	**0.8895 (0.0005)**	**0.8522 (0.0014)**
Corn	−0.0617 (0.9017)	−0.4090 (0.2385)
Japanese green pepper	**0.7494 (0.0094)**	−0.5793 (0.0705)
Red pepper	0.5397 (0.0789)	−0.1765 (0.6902)

a Mean (*n *=* *3) internal temperature at 30 s intervals between 0 and 2 (onion and Japanese green pepper) or 2.5 min (corn and red pepper) of hot plate heating at 250°C was used to measure correlation.

b Significant Pearson correlation coefficients (*p *≤* *.05) are denoted by bold font.

To evaluate whether the changes in DPPH RSA may be related to the formation of Maillard reaction products, the development of brown color in vegetable toppings throughout alternative heat treatment was monitored indirectly using the CIELAB color parameters. Of the three CIELAB color parameters, the L* (i.e., darkness) and a* (redness) parameters are most typically associated with browning (Amrein, Limacher, Conde‐Petit, Amadò, & Escher, 2006). In this study, all vegetable toppings significantly decreased in brightness (i.e., decreasing L* values) with increasing time spent on the hot plate, the largest change of which was observed in onion samples (Table 2). Onion and corn also exhibited slight increases in redness (i.e., increasing a* value) throughout heating, while the redness appeared to remain stable or decrease during the heating of Japanese green pepper and red pepper, respectively.

**Table 2:** CIELAB color parameters (L*, a*, and b*) of alternatively heat‐treated (i.e., hot plate heated) and conventional onion, corn, Japanese green pepper, and red pepper

Sample characteristics		CIELAB color parameters^a^		
Vegetable toppings	Heating time^b^ (min)	L*	a*	b*
Onion	0	85.8 ± 0.1^a^	−2.5 ± 0.8^e^	9.7 ± 0.2^d^
	0.5	83.3 ± 0.8^b^	−1.9 ± 0.1^d^	11.2 ± 0.5^c^
	1.0	81.4 ± 0.6^b^	−0.5 ± 0.3^c^	12.7 ± 0.8^b^
	1.5	77.3 ± 1.0^c^	1.3 ± 0.2^b^	15.0 ± 0.7^a^
	2.0	73.0 ± 1.0^d^	3.0 ± 0.3^a^	15.8 ± 0.2^a^
	3.0 (steam)	83.3 ± 0.4^b^	−3.0 ± 0.1^e^	10.6 ± 0.2^cd^
Corn	0	75.8 ± 0.6^a^	2.6 ± 0.4^bc^	26.5 ± 1.5^bc^
	0.5	75.8 ± 0.6^a^	3.5 ± 0.2^ab^	27.7 ± 0.2^ab^
	1.0	75.0 ± 0.7^ab^	3.9 ± 0.3^a^	28.9 ± 0.4^a^
	1.5	73.5 ± 0.5^bc^	3.4 ± 0.3^bc^	26.7 ± 0.7^bc^
	2.0	73.3 ± 0.5^c^	3.6 ± 0.2^bc^	25.8 ± 0.3^bc^
	2.5	72.8 ± 0.4^c^	3.6 ± 0.2^c^	25.1 ± 0.3^c^
Japanese green pepper	0	65.6 ± 0.3^a^	−5.4 ± 0.0^a^	15.4 ± 0.3^b^
	0.5	65.1 ± 0.6^a^	−6.1 ± 0.1^b^	16.3 ± 0.1^ab^
	1.0	64.7 ± 0.9^ab^	−6.4 ± 0.4^b^	16.3 ± 1.7^ab^
	1.5	63.0 ± 0.3^bc^	−5.3 ± 0.0^a^	14.4 ± 0.1^b^
	2.0	62.3 ± 1.3^c^	−5.1 ± 0.3^a^	17.9 ± 1.0^a^
	3.0 (steam)	63.8 ± 0.1^abc^	−7.6 ± 0.1^c^	18.5 ± 0.0^a^
Red pepper	0	59.6 ± 0.9^ab^	25.3 ± 0.2^a^	18.6 ± 0.3^ab^
	0.5	59.2 ± 0.4^b^	24.6 ± 0.2^ab^	18.1 ± 0.5^ab^
	1.0	59.6 ± 0.5^ab^	24.5 ± 0.3^ab^	19.6 ± 0.5^a^
	1.5	59.0 ± 0.6^b^	23.4 ± 0.3^c^	18.5 ± 0.9^ab^
	2.0	58.9 ± 0.5^b^	21.4 ± 0.3^d^	16.1 ± 0.4^cd^
	2.5	58.1 ± 0.9^b^	19.5 ± 0.5^e^	14.8 ± 0.5^d^
	3.0 (steam)	61.2 ± 0.7^a^	23.6 ± 0.7^bc^	17.5 ± 0.8^bc^

a For each vegetable topping, means in the same column not sharing similar superscript letters are significantly different as determined by Tukey's honestly significant difference test (*p *≤* *.05).

b Individual samples of vegetable topping (15 g fresh weight) were heated on a hot plate (250°C) without agitation. Conventional samples for onion and pepper samples were produced by steam blanching for 3 min.

Of the vegetables assessed, the only significant correlation between both L* and a* parameters and DPPH RSA was observed in onion samples (Table 3). The development of brown color in onion under heated environments has been attributed to the formation of advanced Maillard reaction products such as melanoidins, which also exhibit antioxidant capacity (Moreno, Corzo‐Martínez, del Castillo, & Villamiel, 2006). This suggests that the development of Maillard reaction products may have contributed to the sharp increase in DPPH RSA during hot plate heating (Figure 2). In contrast, the development of brown color in heated pepper and corn samples was not associated with changes in DPPH RSA (Table 3). These findings further demonstrate that not only are the effects of heating on overall antioxidant capacity vegetable dependent, but are the result of complex chemical changes involving both inherent and newly formed antioxidants.

**Table 3:** Pearson correlation coefficients and their significance between CIELAB color parameters L*, a*, and b* and DPPH radical scavenging activity (μmol Teq/g dw) during alternative heat treatment (i.e., hot plate heating) of onion, corn, Japanese green pepper, and red pepper

Vegetable toppings	Pearson correlation coefficients between CIELAB color parameters^a^ and DPPH radical scavenging activity (*p*‐value)		
	L*	a*	b*
Onion	−**0.9767 (0.0000)**	**0.9802 (0.0000)**	**0.9228 (0.0000)**
Corn	**0.5401 (0.0002)**	−0.0558 (0.8162)	0.1820 (0.3865)
Japanese green pepper	0.2414 (0.2719)	0.0948 (0.7118)	0.0221 (0.9363)
Red pepper	0.1403 (0.5230)	−0.0717 (0.7613)	−0.2844 (0.1315)

a Color measurements were performed on lyophilized and ground vegetable topping samples that were alternatively heat‐treated (i.e., hot plate heating) at 30 s intervals between 0 and 2 (onion and Japanese green pepper) or 2.5 min (corn and red pepper). Significant Pearson correlation coefficients (*p *≤* *.05) are denoted by bold font.

**Figure 3:**
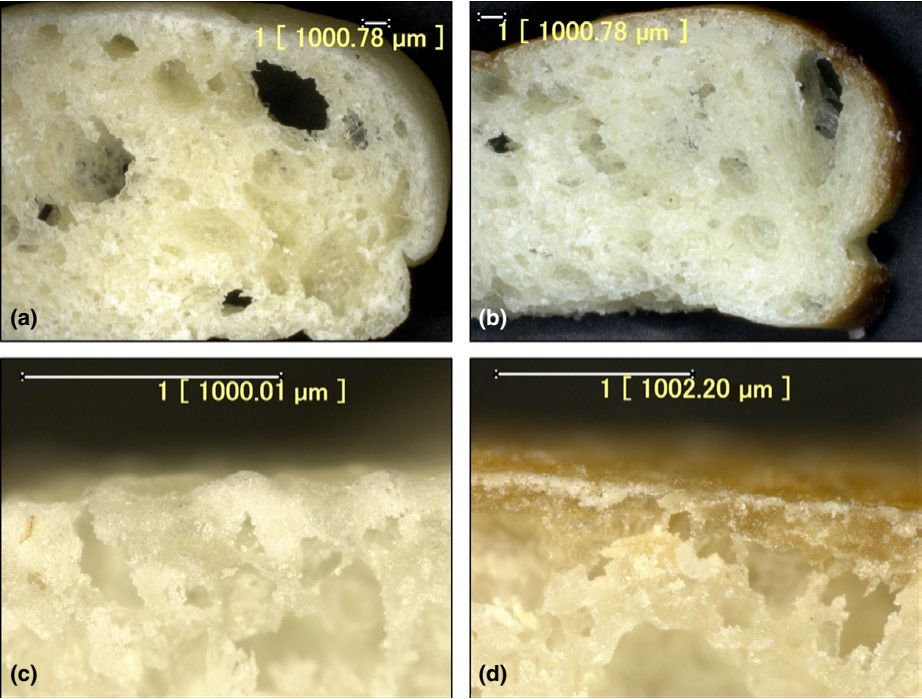
Cross section and crust of conventional par‐baked pizza base (a and c, respectively) and alternatively heat‐treated (i.e., additional heating at 500°C for 50 s) pizza base (b and d, respectively). Scales for each image are as indicated

AHT pizza base was prepared by baking commercially available par‐baked pizza base for 50 s at 500°C. These conditions were found in preliminary experiments to induce maximum surface browning with minimal changes in internal crumb structure, characteristics of which were monitored by visual comparison of cross‐sectional images of pizza bases pre‐ and post‐alternative heat treatment (Figure 3). It can be observed that after the subsequent heat treatment, AHT pizza bases developed a thin, brown crust on the top and side surfaces of the pizza base.

Higher crust and crumb TPC were observed in AHT pizza base than in the conventional product (Table 4). The higher TPC in AHT pizza base may be due to the dissociation of some polyphenols from the complexes they formed with other components of bread, such as bread proteins or free amino acids, during the initial par‐baking step (Świeca et al., 2013). Maillard reaction products formed may also interfere with the Folin–Ciocalteu assay and potentially result in erroneous measurements of TPC in breads (Michalska et al., 2008). This may explain the larger deviation in TPC among replicates of AHT crust samples, which were the only samples to undergo browning and therefore were most susceptible to have interfering compounds present during measurement.

**Table 4:** Total polyphenol content and DPPH radical scavenging activity of conventional and alternatively heat‐treated (i.e., baking at 500°C for 50 s) par‐baked pizza base^a^

Components	Total polyphenol content (mg GAE/g dw)		DPPH antioxidant capacity (μmol Teq/g dw)	
	Conventional	Alternative heat treatment	Conventional	Alternative heat treatment
Surface (crust)	2.03 ± 0.07^a^	2.35 ± 0.19^a^	0.41 ± 0.01^b^	1.20 ± 0.05^a^
Crumb	1.95 ± 0.08^b^	2.32 ± 0.05^a^	0.44 ± 0.02^a^	0.47 ± 0.01^a^
Total	1.74 ± 0.08^a^	1.84 ± 0.07^a^	0.42 ± 0.01^b^	0.67 ± 0.05^a^

a For each component, means (*n *=* *3) between conventional and alternatively heat‐treated par‐baked pizza base not followed by similar superscript letters are significantly different as determined by a *t*‐test assuming equal variances.

The overall DPPH RSA of AHT pizza base was significantly higher than that of the conventional product due to increased surface DPPH RSA (Table 4). Although the higher DPPH RSA in AHT crust may be associated with the higher TPC observed, the similar DPPH RSA of conventional and AHT crumb despite higher TPC in the latter suggest that other antioxidants were contributing to the higher DPPH RSA. A more likely explanation of the higher DPPH RSA in AHT crust is the formation of Maillard reaction products during heating (Michalska et al., 2008; Moore et al., 2009; Morales et al., 2009). As pizza bases account for a majority of the final, frozen pizza by weight and may act as a source of Maillard reaction products, AHT pizza base has potential use as a medium for increasing the *in vitro* antioxidant capacity of the final product.

### 3.2 Commercial applicability of AHT ingredients

**Figure 4:**
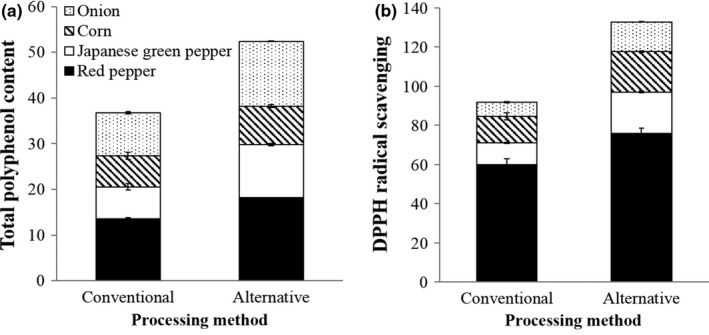
Total polyphenol content (mg gallic acid equivalent per 12 g vegetable topping wet weight) (a) and DPPH radical scavenging activity (μmol Trolox equivalent per 12 g vegetable topping wet weight) (b) of conventional or alternatively heated (i.e., hot plate heated) onion, corn, Japanese green pepper, and red pepper after reheating on the corresponding pizza base (*n *=* *3). Fill patterns for each ingredient are depicted as indicated

The TPC and DPPH RSA of conventional and AHT vegetable toppings after reheating as part of an assembled pizza are shown in Figure 4. Based on a starting weight of 12 g per vegetable topping, the use of AHT samples instead of conventional samples would increase TPC by an estimated 1.5‐, 1.2‐, 1.6‐, and 1.3‐fold, respectively, for onion, corn, Japanese green pepper, and red pepper in reheated pizzas. Similarly, DPPH RSA was estimated to increase by 2.1‐, 1.5‐, 1.9‐, and 1.3‐fold, respectively. These values were estimated using the moisture content of onion, corn, Japanese green, and red pepper, which were 90.7 ± 0.0, 77.8 ± 0.2, 93.6 ± 0.1, and 91.8 ± 0.1%, respectively, for conventional toppings and 87.8 ± 0.1, 73.6 ± 0.6, 91.9 ± 0.2, and 89.9 ± 0.1%, respectively, for AHT toppings. Evidently, AHT vegetable toppings had slightly lower moisture contents than their conventional counterparts. Therefore, it should be noted that the higher TPC and DPPH RSA of AHT samples were in part due to these samples having lower moisture content. For example, 12 g of the former would contain more solid content, and presumably more antioxidants, than 12 g of the latter. Nevertheless, these results suggest that the use of AHT vegetable toppings in lieu of conventional ingredients appears to offer the advantage of increasing TPC and DPPH RSA of the final, reheated product.

Smaller differences between conventional and AHT pizza base were observed in terms of TPC (190.0 ± 4.1 and 203.0 ± 10.9 mg GAE per pizza base, respectively) and DPPH RSA (87.0 ± 5.4 and 100.0 ± 4.1 μmol Teq per pizza base, respectively). Based on these results, the AHT pizza base produced in this study may not be a viable medium for increasing the potential antioxidant capacity of processed, frozen pizza, despite having higher DPPH RSA than the conventional pizza base prior to reheating. The smaller difference between DPPH RSA of AHT and conventional pizza base may be the result of Maillard reaction products forming in the latter upon reheating.

The commercial applicability of AHT vegetable toppings was also assessed based on their sensory properties, the results of which are shown in Table 5. Sensory evaluation was not conducted on AHT pizza base due to its TPC and DPPH RSA values being similar to conventional pizza bases. Significant differences (*p *≤* *.05) were not observed for any attribute between conventional and AHT samples of any vegetable topping material. Near significant (i.e., *p *<* *.10) differences were found for the attributes of two vegetable toppings, wherein the appearance of conventional onion (*p *=* *.067) and aroma of AHT Japanese green pepper (*p *=* *.096) were more preferred than their respective counterparts. These results suggest that, when included as part of a processed pizza product, the acceptability of AHT vegetable toppings was comparable to their conventional counterparts.

**Table 5:** Acceptability^a^ of sensory attributes of conventional and alternatively heat‐treated (i.e., hot plate heated) vegetable toppings as an ingredient of frozen pizza after reheating (230°C for 10 min)

Sensory attribute	Vegetable toppings							
	Onion		Corn		Japanese green pepper		Red pepper	
	Conventional	Alternative heat treatment	Conventional	Alternative heat treatment	Conventional	Alternative heat treatment	Conventional	Alternative heat treatment
Appearance	6.5 ± 1.1	5.9 ± 1.0	6.5 ± 1.4	6.0 ± 1.5	5.3 ± 1.6	5.0 ± 1.4	5.6 ± 1.4	5.6 ± 2.0
Taste	6.7 ± 1.3	6.5 ± 1.9	6.5 ± 1.3	6.5 ± 1.7	6.1 ± 1.3	5.9 ± 1.8	5.9 ± 1.4	6.2 ± 1.5
Aroma	6.6 ± 1.2	6.3 ± 1.3	6.2 ± 1.2	6.2 ± 1.2	5.8 ± 1.2	6.3 ± 1.3	5.9 ± 1.4	6.6 ± 1.5
Texture	6.0 ± 1.8	5.8 ± 1.5	5.6 ± 2.0	6.3 ± 1.7	5.7 ± 1.3	6.2 ± 1.7	6.6 ± 1.5	6.5 ± 1.0
Overall	6.2 ± 1.3	6.0 ± 1.6	6.0 ± 1.5	6.3 ± 1.5	5.5 ± 1.1	5.7 ± 1.7	5.7 ± 1.3	6.3 ± 1.3

a For each sensory attribute, means (*n *=* *11) between conventional and alternatively heat‐treated samples of each ingredient were compared using a paired *t*‐test. Significant differences (*p *≤* *.05) were not observed.

In this study, AHT by hot plate heating appears to have potential as a method for increasing the TPC and DPPH RSA of selected vegetable toppings used in frozen pizza with minimal effects on acceptability. Future studies should focus on exploring other heating methods and optimal heating conditions for the purpose of improving conventional heating methods, and evaluate the *in vivo* benefits of the observed increases in *in vitro* antioxidant capacity. While Maillard reaction products formed during the high temperature heating of foods may potentially have health‐enhancing effects *in vivo* (Patrignani et al., 2016), toxicants such as acrylamide and furanic compounds may also form. Although the formation of such toxicants may be effectively mitigated by nonconventional heating methods such as a combination of radiofrequency and conventional baking (Anese, Manzocco, Calligaris, & Nicoli, 2013), this simultaneous development of antioxidative compounds and toxicants causes the risk‐benefit assessment of heating on biological healthfulness to be a difficult task (Morales et al., 2009). Therefore, although hot plate heating was selected as an alternative heating method for producing AHT vegetable toppings in the current study, other heating methods with less risk of producing toxicants should be investigated.

## 4 CONCLUSION

The results of this study demonstrated that selected ingredients of processed pizza produced using nonconventional heating methods can have higher total polyphenol content (TPC) and DPPH radical scavenging activity (DPPH RSA) than conventional ingredients, and may be incorporated into pizza with minimal changes in consumer acceptability. In particular, replacing conventional vegetable toppings with alternatively heat‐treated (i.e., hot plate heated) counterparts increased total polyphenol content (TPC) by 1.2‐fold to 1.6‐fold and DPPH radical scavenging activity (DPPH RSA) by 1.3‐fold to 2.1‐fold in the reheated product. On the other hand, replacing conventional pizza base with the alternatively heat‐treated pizza base prepared in the current study (i.e., commercially available par‐baked pizza base heated for an additional 50 s at 500°C) only increased TPC and DPPH RSA by 1.1‐fold, despite the latter having undergone more Maillard browning and exhibiting higher *in vitro* antioxidant capacity before reheating. Based on these findings, simply altering the heating conditions applied during the processing of individual ingredients to facilitate the dissolution or formation of antioxidative compounds could potentially increase the *in vitro* antioxidant capacity of the final, assembled, and reheated product. This method is advantageous over existing strategies for increasing potential healthfulness, such as the reformulation or enrichment of raw ingredients, which may lead to undesirable changes to the product. Although various factors such as the *in vivo* benefits and production of carcinogenic byproducts should be considered when applying such strategies, the use of alternative heat treatments when preparing food ingredients may be a viable method for increasing the potential healthfulness of frozen pizza and warrant further study.

## CONFLICT OF INTEREST

The authors declare that there are no conflict of interests in relation to this study.

## ETHICAL REVIEW

Human or animal testing was not conducted in relation to this study.

## INFORMED CONSENT

Verbal consent was obtained from all panelists involved in the sensory evaluation.
